# Controllable Growth of Ordered In-Plane Ge Hut Wires on Trench-Patterned Si Substrate

**DOI:** 10.3390/nano16070423

**Published:** 2026-03-31

**Authors:** Fei Gao, Ming Ming, Jie-Yin Zhang, Jian-Jun Zhang

**Affiliations:** 1 Department of Physics, Qilu Institute of Technology, Jinan 250200, China; 2 Beijing National Laboratory for Condensed Matter Physics, Institute of Physics, Chinese Academy of Sciences, Beijing 100190, China; mingming@sslab.org.cn (M.M.); zhangjieyin@sslab.org.cn (J.-Y.Z.); jjzhang@iphy.ac.cn (J.-J.Z.); 3 Songshan Lake Materials Laboratory, Dongguan 523808, China

**Keywords:** size-controllable, germanium hut wires, epitaxial growth

## Abstract

The controllable growth of in-plane Ge nanowires provides alternative material foundations for the scalability of Ge-based semiconductor qubit devices. Here, ordered in-plane Ge hut wires with controllable size are grown on the trench-patterned Si substrate by molecular beam epitaxy. By tuning the thickness of the SiGe alloy layer, which acts as strain buffered layer, GeSi mounds with controllable size are achieved. Subsequently, through the deposition of a Ge layer followed by in situ annealing, we realize the size-controllable growth of the Ge nanowire with a height from 1.8 nm to 4.0 nm, as characterized by AFM and TEM techniques. These size-tunable and catalyst-free Ge hut wires provide a promising pathway toward the fabrication of integrated nanowire-based quantum devices.

## 1. Introduction

Group IV nanomaterials have attracted great attention due to their superior characteristics in semiconductor quantum computation [1]. Group IV materials have advantages in terms of spin decoherence time over Group III–V materials because of the lower hyperfine interactions and a high level of zero-nuclear-spin isotopes [2]. Germanium nanowires as a kind of high-quality Group IV material platform have become a strong contender for hole-spin qubit owing to the high hole mobility, lower hyperfine interactions compared to electron spin, strong spin-orbit interactions, good ohmic contact with metal electrodes, and good CMOS compatibility [3,4,5,6]. Some impressive achievements based on germanium nanowire devices have been obtained including the demonstration of electrically driven spin qubits [7], the coupling of a hole charge to a superconducting resonator [8], ultrafast and electrically tunable Rabi frequency exceeding 1.2 GHz [9], and a hard superconducting gap [10].

All aforementioned achievements are based on the rapid development of Ge nanowire growth technology. Compared to the top-down nanowire etching method [11], Ge/Si core-shell nanowires synthesized by a Vapor–Liquid–Solid (VLS) mechanism have higher quality [12], such as a smooth surface, small dimensions, and a higher hole mobility [13]. At the same time, the diameter of the Ge/Si core-shell nanowires can be conveniently controlled by adjusting the size of the metal catalyst. But the metal catalyst is detrimental to the semiconductor nanowires. An in-plane surface epitaxy technique reported in ref. [14] can realize the in-plane lateral epitaxy of the ZnO nanocrystal, which is influenced by a crystalline state of the substrate surface layer defined as a “surface skin effect”. This powerful nano-epitaxy method allows for the spatial control over the properties and function of low-dimensional heterostructures. A self-assembled growth technique—molecular beam epitaxy (MBE)—is also employed to achieve the in-plane growth of Ge hut wires on a Si substrate [15]. These in-plane nanostructures can avoid the issues of metal contamination, transfer difficulties, inhomogeneity due to VLS memory effects, and elemental segregation associated with the VLS method [16], enhancing compatibility with CMOS device fabrication processes. In addition to experimental methods, theoretical modeling such as density functional theory (DFT) can provide valuable insights into the growth mechanisms of semiconductor nanowires [17]. In recent years, we have successfully demonstrated the self-assembled growth of ordered Ge(Si) hut wires with controllable position, distance, length, and a high mobility, laying a foundation for the integration and addressing of semiconductor qubit devices based on Ge nanowires [18,19,20,21]. However, realizing the size control of the in-plane (105) faceted Ge hut wires remains a challenge.

In this work, combining the nano-fabrication and MBE growth technique, the size control of the in-plane ordered Ge hut wires is achieved by adjusting the structure of the SiGe mound and by the subsequent deposition of Ge layers on this mound. Atomic force microscopy (AFM) and transmission electron microscopy (TEM) characterizations are utilized to demonstrate the size and the high quality of the Ge hut wires. Based on our previous work reported in Ref. [18], in which we achieved the controllable growth of in-plane (105) faceted Ge hut wires in regards to position and length, this work further demonstrates the size-controllable growth of these Ge hut wires. Different from the site-selective growth approach reported in Ref. [21], the size controllability achieved here is realized via a GeSi mound-mediated mechanism.

## 2. Materials and Methods

**Figure 1:**
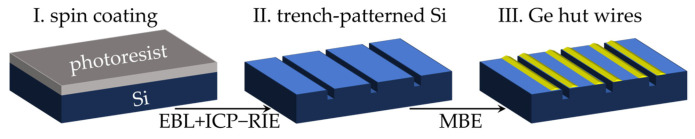
Schematic diagram of the trench-patterned Si substrate preparation and the growth of the Ge hut wires. Blue represents the Si substrate, and yellow represents the Ge hut wires.

A 4-inch trench-patterned Si (001) wafer is fabricated according to the following steps. After spin-coating a photoresist (AR-P6200.09, Allresist GmbH, Strausberg, Germany) with a thickness of 300 nm onto the Si substrate, it is baked on a hotplate at 180 °C for 3 min to enhance adhesion. Then it is loaded into the electron beam lithography system (EBL, JBX-6300FS, JEOL, Tokyo, Japan) to undergo the process of electron beam patterning. Subsequently, by developing the photoresist, post-exposure baking at 130 °C for 3 min, etching in the ICP-RIE (Inductively Coupled Plasma Reactive Ion Etching, PlasmaPro100Cobra, Oxford Instruments, Bristol, UK) system, and finally removing the photoresist in RIE system, we obtain the trench structures along the [100] crystal direction with a depth of ~70 nm and a width of ~100 nm. Figure 1 shows the brief flow of the substrate preparation and sample growth processes.

The trench-patterned Si substrate is used to deposit a Si and Ge layer by MBE to study the size control of the in-plane ordered Ge hut wires. The 4-inch Si substrate is cleaved into 10 × 10 mm^2^ pieces. Then, the 10 × 10 mm^2^ sample is loaded into the MBE system (Octoplus 500 EBV, MBE-Komponenten, Weil der Stadt, Stuttgart, Germany) after dipping in a 5% HF solution for the deoxidation and hydrogen passivation. Next, the small Si substrate is heated to 400 °C for 1 h for degassing and then to 650 °C for 10 min for dehydrogenation. Finally, after the growth of a 60 nm Si buffer layer at 450 °C, Ge hut wires are grown along the edges of the trenches first by the deposition of a 2 to 7 nm Si_67_Ge_33_ layer at 560 °C with Ge growth of 0.1 Å/s, and then by the deposition of a 4 to 7 Å Ge layer at 560 °C with a rate of 0.07 Å/s, followed by in situ annealing at 560 °C for 15 min.

AFM system (Bruker Multimode 8, Bruker, CA, USA, tapping mode) and TEM equipment (JEOL 2100 plus, JEOL, Tokyo, Japan, operating at 200 kV) are used to characterize the surface morphology and cross-section of the Ge hut wires, respectively.

## 3. Results and Discussion

### 3.1. Gesi Mound

**Figure 2:**
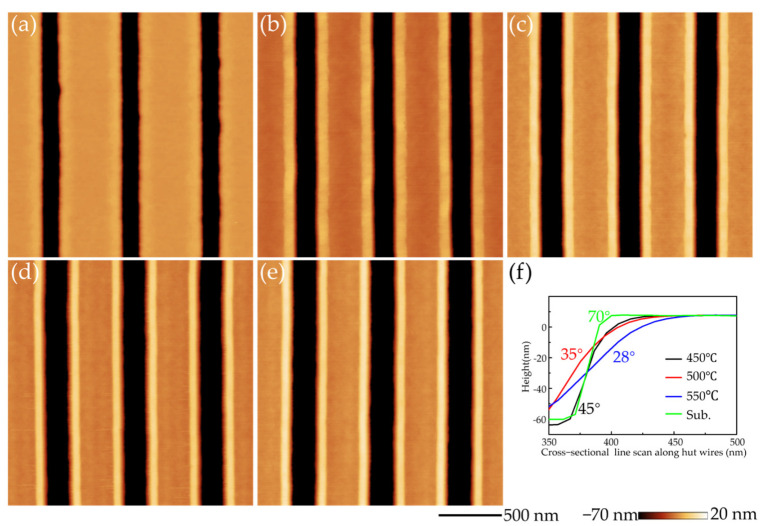
AFM images of (**a**) 60 nm Si buffer layer. (**b**–**e**) Ordered in-plane GeSi mounds with SiGe layer thicknesses of 2 nm, 3 nm, 5 nm, and 7 nm, respectively. (**f**) The AFM line-scans perpendicular to the trenches after the growth of Si buffer layer at different growth temperatures.

On patterned Si substrates, previous theoretical calculations showed that SiGe nanostructures preferentially form on the pattern edges or inside the pits, depending on the pattern’s inclination angle [22]. For large inclination angles, nucleation on the edges is favored for better strain relaxation. However, for small inclination angles, nucleation inside the pits is energetically favorable. The patterned trenches have an inclination angle of about 70° (Figure 2a). After a 60 nm thick Si buffer layer is grown, the sidewall pattern evolves into a shallow angle. The higher the growth temperature of the Si buffer layer, the smaller the inclination angle. The Si-buffer-layer growth was performed at 450 °C, 500 °C, and 550 °C, respectively. Correspondingly, as shown by AFM line scans in Figure 2f, inclination angles evolved to about 45°, 35°, and 28°. In addition, lower growth temperatures such as 400 °C lead to serrated defects on the trench sidewall. To guarantee the preferential growth on the edges, the Si buffer growth is performed at 450 °C for the subsequent SiGe growth.

After the Si buffer layer, Si_67_Ge_33_ alloy layers with different thicknesses of 2 nm, 3 nm, 5 nm, and 7 nm were deposited at 560 °C on Si substrates followed by in situ annealing at 560 °C for 6 min. As shown in Figure 2b–e, ordered GeSi mounds formed on the edges of the trenches. It shows that the deposition of Si_67_Ge_33_ alloy layers from 3 to 7 nm results in GeSi mounds with well-defined surface morphology. We note that the 6 min annealing can ensure that the Si and Ge adatoms have sufficient diffusion time, thus significantly improving the mound surface morphology and changing it from discontinuous to continuous one-dimensional structure. But if the thickness of the SiGe layer is only 2 nm, the surface of the mound exhibits discontinuities, indicating that 2 nm is still below the critical thickness.

**Figure 3:**
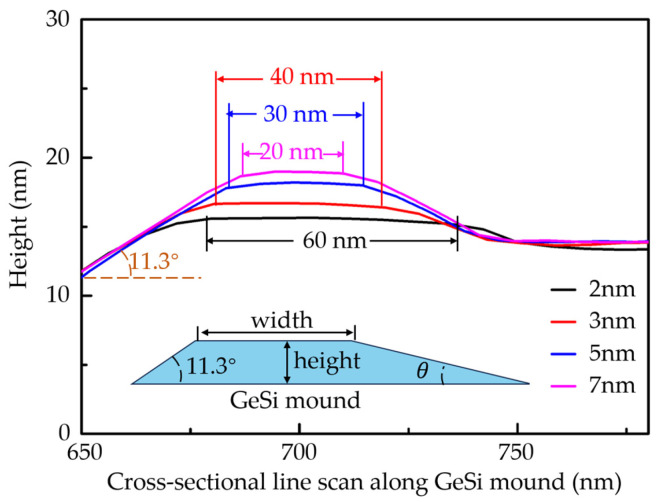
AFM line scan along the cross-section of GeSi mound grown on the trench-patterned Si substrate. The inset picture is the cross-sectional schematic diagram of the GeSi mound.

To illustrate the evolution of the cross-sectional morphology of the GeSi mounds with increasing Si_67_Ge_33_ alloy layer thickness from 2 nm to 7 nm, we performed an AFM line-scan perpendicular to trenches, as shown in Figure 3. The inset picture is the cross-sectional schematic diagram of the GeSi mound, showing its asymmetric trapezoidal geometry. We find that the inclination angle on the side of the mound next to the trench remains constant at 11.3°, which means that the surface on that side is bounded by (105) facets. It should be noted that the upper sidewall inclination angle of the trench can evolve into a stable (105) facet with the growth of the Si_67_Ge_33_ alloy layer [18], due to the low surface energy of the (105) facet and the [100]-oriented trench structure. This can induce the formation of the (105) facet on the side of the mound adjacent to the trench. In contrast, the sidewall inclination angle of the mound away from the trench increases gradually with the Si_67_Ge_33_-layer thickness and remains smaller than 11.3°. The purple, blue, and red lines in Figure 3 show that even though the Si_67_Ge_33_ layer thicknesses are different, the base size of the SiGe mound is essentially the same, about 70 nm, owing to the consistent growth temperature and Ge concentration [23]. This stability in the mound structure enables controllable and uniform nanowire dimensions. Furthermore, due to the low surface energy of the (105) facet, the sidewall inclination angle of the mound away from the trench increases with the Si_67_Ge_33_-layer thickness, reaching up to 11.3°. GeSi mounds form via a nucleationless process. This process originates from the inherent morphological instability of the strained Si_67_Ge_33_ alloy film during heteroepitaxy on the patterned Si substrate. Such nucleationless growth is essential for the formation of ordered array structures [24].

We can conclude from Figure 3 that the width of the top platform of the mound decreases from about 60 nm to about 20 nm and the height increases from about 2 nm to about 5 nm as the SiGe layer thickness increases from 2 nm to 7 nm. In other words, the mound size can be controlled by simply adjusting the SiGe layer thickness. Such a GeSi mound serves as a strained buffer layer for the subsequent growth of Ge hut wires on its platform. The width of the mound top platform determines the size of the Ge hut wire. Moreover, the position-controllable growth of the mound enables the ordered growth of the Ge hut wires. In addition, the mound can grow in height as the deposition thickness continues to increase, bounded by two (105) facets.

### 3.2. Ge Hut Wires

**Figure 4:**
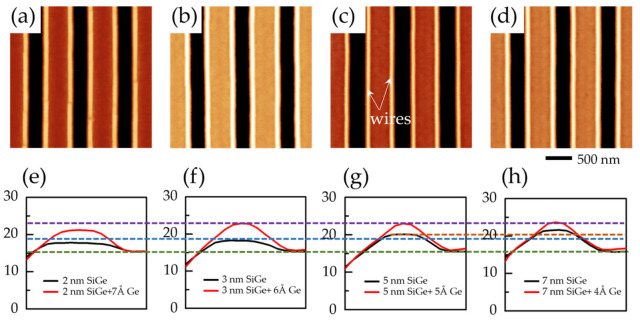
(**a**–**d**) AFM images of the ordered in-plane Ge hut wires after the growth of 7 Å, 6 Å, 5 Å, and 4 Å Ge on the GeSi mounds. (**e**–**h**) The red lines are the AFM line-scans across the Ge hut wire structures in (**a**–**d**), respectively. The black lines are the AFM line scans across the corresponding GeSi mounds. The green, blue, purple and orange dashed lines are used to emphasize the height difference of these Ge hut wires.

Once GeSi mounds form on the Si substrate, Ge hut wires will nucleate on top of the GeSi mounds following the growth of a Ge layer and subsequent in situ annealing. The misfit strain between the Ge and GeSi mounds is smaller than the strain between Ge and Si. Therefore, Ge hut wires prefer to nucleate on the GeSi mounds rather than on the flat surface owing to the lower diffusion barriers [18] and the better strain relaxation. Figure 4a–d show AFM images of the Ge hut wires after the growth of 7 Å, 6 Å, 5 Å, and 4 Å Ge on GeSi mounds with corresponding GeSi layer thicknesses of 2 nm, 3 nm, 5 nm, and 7 nm, respectively. Corresponding cross-sectional line scans of Ge hut wire in Figure 4a–d are shown in Figure 4e–h, respectively. In Figure 4a, the Ge hut structures exhibit discontinuous surfaces due to the discontinuity of the underlying GeSi mound surfaces. Correspondingly, Figure 4e shows that the GeSi mound cannot evolve into a (105)-faceted Ge hut wire with a triangular cross-section.

Figure 4b–d show that ordered in-plane Ge hut wires form on the different SiGe mounds. The Ge hut wires, bounded by (105) facets, have a triangular cross-section with an aspect ratio of 1:10. Since Ge hut wires are grown on the top plateau of the GeSi mound, the width of the top platform determines the height of the Ge hut wires. From Figure 3 and Figure 4f–h, we can conclude that the height of the Ge hut wires decreases from 4 nm to 2 nm as the width of the top plateau decreases from 40 nm to 20 nm. In Table 1, we have also clearly listed the specific growth parameters of the Ge hut wires shown in Figure 4, along with the relevant parameter information for the GeSi mounds and Ge hut wires determined from the AFM images.

**Figure 5:**
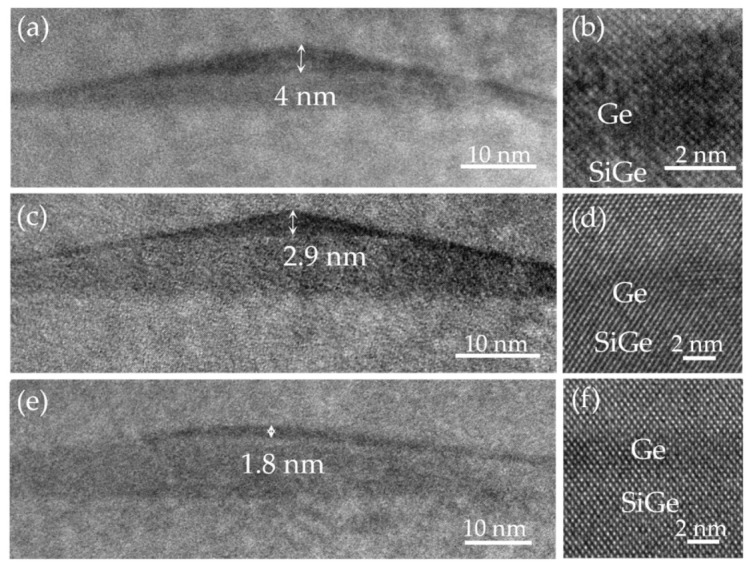
The TEM images of the Ge hut wires with different height. The growth parameters are (**a**) 6 Å Ge on a 3 nm SiGe layer, (**c**) 5 Å Ge on a 5 nm SiGe layer, (**e**) 4 Å Ge on a 7 nm SiGe layer, respectively. (**b**), (**d**) and (**f**) are the corresponding partially magnified TEM images in (**a**), (**c**), and (**e**), respectively.

To confirm our results, we also characterized the cross-sectional morphology of the Ge hut wires in Figure 4b–d by TEM, with the results shown in Figure 5a–f. The TEM images in Figure 5a,c,e present the triangular cross-sectional morphology of Ge hut wires, with heights of 4 nm, 2.9 nm, and 1.8 nm, respectively. They also confirm that the Ge hut wires are definitely located on the platform of the GeSi mound. These TEM results are consistent with the AFM results, further providing strong validation of our conjecture. Figure 5b,d,f show the partially magnified TEM images in (a), (c), and (e), respectively. No defects are observable in these magnified TEM images, demonstrating the perfect crystallinity of the Ge hut wires. In conclusion, we have achieved the size control of ordered in-plane Ge hut wires on trench-patterned Si substrate by tuning the width of the top platform of the GeSi mounds.

## 4. Conclusions

In summary, we have conducted a study on the size-controllable growth of the ordered in-plane Ge hut wires on a trench-patterned Si substrate. The lower growth temperature of the Si buffer layer provided favorable conditions for the formation of GeSi mounds at the trench edges. The height and the width of the top plateau of the GeSi mounds can be easily tuned by varying the deposition thickness of the SiGe layer. Ordered in-plane Ge hut wires with tunable heights from 1.8 nm to 4 nm have been achieved by the deposition of a Ge layer on the GeSi mounds followed by in situ annealing.

These Ge hut wires can serve as an excellent platform for investigating the influence of nanowire size on their optical properties through photoluminescence spectroscopy. The catalyst-free, ordered and size-controllable in-plane Ge hut wires also provide a platform for the Ge quantum dot qubits. In such Ge hole qubit devices, the small dimension of the Ge hut wires enables strong quantum confinement and discrete energy levels essential for qubit operation. Moreover, the in-plane and site-controlled properties of the Ge hut wires are favorable for the large-scale integration, which is important for quantum computing architectures.

## Figures and Tables

**Table 1:** Detailed parameters of GeSi mounds and Ge hut wires in Figure 3.

	Growth Parameter	Height of GeSi Mounds	Top Plateau of GeSi Mounds	Height of Ge Hut Wires
Figure 4a	2 nm SiGe + 7 Å Ge	2 nm	60 nm	No wires formed
Figure 4b	3 nm SiGe + 6 Å Ge	3 nm	40 nm	4 nm
Figure 4c	5 nm SiGe + 5 Å Ge	4 nm	30 nm	3 nm
Figure 4d	7 nm SiGe + 4 Å Ge	5 nm	20 nm	2 nm

## Data Availability

The original contributions presented in this study are included in the article. Further inquiries can be directed to the corresponding author.
